# Selection signatures in Canchim beef cattle

**DOI:** 10.1186/s40104-016-0089-5

**Published:** 2016-05-05

**Authors:** Ismael Urbinati, Nedenia Bonvino Stafuzza, Marcos Túlio Oliveira, Tatiane Cristina Seleguim Chud, Roberto Hiroshi Higa, Luciana Correia de Almeida Regitano, Maurício Mello de Alencar, Marcos Eli Buzanskas, Danísio Prado Munari

**Affiliations:** Departamento de Ciências Exatas, - Univ Estadual Paulista, Faculdade de Ciências Agrárias e Veterinárias, Jaboticabal, São Paulo 14884-900 Brazil; Departamento de Tecnologia, UNESP - Univ Estadual Paulista, Faculdade de Ciências Agrárias e Veterinárias, Jaboticabal, São Paulo 14884-900 Brazil; Embrapa Agricultural Informatics, Campinas, São Paulo 13083-886 Brazil; Embrapa Southeast Livestock, São Carlos, São Paulo 13560-970 Brazil

**Keywords:** Composite breed, Extended haplotype homozygosity, Genomics, Single nucleotide polymorphism

## Abstract

**Background:**

Recent technological advances in genomics have allowed the genotyping of cattle through single nucleotide polymorphism (SNP) panels. High-density SNP panels possess greater genome coverage and are useful for the identification of conserved regions of the genome due to selection, known as selection signatures (SS). The SS are detectable by different methods, such as the extended haplotype homozygosity (EHH); and the integrated haplotype score (iHS), which is derived from the EHH. The aim of this study was to identify SS regions in Canchim cattle (composite breed), genotyped with high-density SNP panel.

**Results:**

A total of 687,655 SNP markers and 396 samples remained for SS analysis after the genotype quality control. The iHS statistic for each marker was transformed into piHS for better interpretation of the results. Chromosomes BTA5 and BTA14 showed piHS > 5, with 39 and nine statistically significant SNPs (*P* < 0.00001), respectively. For the candidate selection regions, iHS values were computed across the genome and averaged within non-overlapping windows of 500 Kb. We have identified genes that play an important role in metabolism, melanin biosynthesis (pigmentation), and embryonic and bone development.

**Conclusions:**

The observation of SS indicates that the selection processes performed in Canchim, as well as in the founder breeds (i.e. Charolais), are maintaining specific genomic regions, particularly on BTA5 and BTA14. These selection signatures regions could be associated with Canchim characterization.

**Electronic supplementary material:**

The online version of this article (doi:10.1186/s40104-016-0089-5) contains supplementary material, which is available to authorized users.

## Background

Canchim cattle has excellent meat yield and quality and has perfomed well when raised on natural pastures of Brazil, but it still has a small inventory with 28–30 thousand registered animals and represents about 3 % of the crossbred animals reared in the country [1]. The Canchim breed was developed in the early 1960’s aiming to combine fitness traits from zebu to the higher reproduction efficiency and meat quality from the Charolais breed [2–4]. The genetic makeup of this composite breed was a 62.5 % Charolais and 37.5 % zebu proportion. The genetic group called MA (resulting of mating between Charolais bulls and ½ Canchim + ½ zebu dams) is widely used by Canchim breeders, mainly to expand the genetic basis of the breed and present an expected proportion of 65.6 % Charolais and 34.4 % zebu [5]. Many studies were carried out in this breed, mostly on quantitative genetics [6–10].

Single nucleotide polymorphisms (SNPs) panels are applied in several studies, including selection signatures, in which regions of the genome maintained from generation to generation due to selection are identified. When positive selection occurs, it is expected that some loci adjacent to favorable mutations on a chromosome region segregates in a dependent manner, resulting in high and non-random allelic frequencies in specific regions [11]. The recent positive selection is characterized by increased linkage disequilibrium and decreased genetic variability in the population [12], which is caused by the rapid fixation of mutations favorable to the selected traits [13]. When positively selected alleles achieve high frequency, other alleles in linkage disequilibrium also increase their frequencies (hitchhike), retaining not only the favorable allele, but a region of the genome next to it (selective sweep effect) [14].

The identification of selection signatures could be used to assess the genome regions that control the quantitative traits under selection, and genes associated with phenotypic variations of the traits of interest, allowing to understand the biological mechanisms involved in the phenotypic manifestation. Therefore, the aim of our study was to identify and characterize selection signatures in Canchim using high density SNP data by means of the integrated haplotype score method.

## Methods

### Ethics statement

This study was approved by the Embrapa Southeast Livestock Ethical Committee for Animal Use (CEUA-CPPSE) under protocol 02/2009.

### Animals and genotyping data

Genotypic data on 285 Canchim animals and 114 MA animals were used in this study. The animals were born between 1999 and 2005, being 205 females and 194 males. All animals were genotyped with the Illumina BeadChip BovineHD panel, consisting of 786,799 SNP markers.

According to Mokry et al. [15], the persistence of linkage disequilibrium phase between Canchim and MA genetic group was consistent and high, suggesting that both groups could be considered together in genetic evaluation programs. Thus, we have decided to treat both Canchim and MA as one dataset for further analyses and refer to all of these animals as Canchim.

### Quality control of genotypes and imputation

The genotype quality control (QC) was performed using the snpStats package [16] in *R* statistical software [17]. Only SNPs on autosomal chromosomes with defined position according to the UMD_3.1 bovine genome assembly [18], were used in the analyses. SNPs with genotype calling score below 0.20 were unreliable and coded as missing genotypes. Minor allele frequencies (MAF) below 1 %, call rate for SNP below 95 %, and call rate for samples below 90 % were excluded.

The software BEAGLE v.3.3.2 [19] was used for inference of linkage phase, haplotype construction, and imputation of missing genotypes. These steps are required for the study of selection signatures.

### Identification of selection signatures

The integrated haplotype score (iHS), a statistical methodology proposed by Voight et al. [20] and derived from the extended haplotype homozygosity (EHH) methodology [21], was used to identify the selection signatures. The EHH methodology, also known as long-range haplotype, detects recent positive selection by analyzing the haplotypes structure of individuals in a population. The method accounts for the allelic frequencies and the extent of linkage disequilibrium. Linkage disequilibrium refers to the non-random association between alleles at two loci [22], whereas the EHH methodology measures the association between a single core allele at one locus with multiple loci at different distances.

The integrated haplotype score (iHS) was calculated as:

$$ iHS=\frac{ \ln \left(\frac{iH{H}_A}{iH{H}_D}\right)-E\left[ \ln \left(\frac{iH{H}_A}{iH{H}_D}\right)\right]}{SD\left[ \ln \left(\frac{iH{H}_A}{iH{H}_D}\right)\right]} $$

in which *iHH*_*A*_ and *iHH*_*D*_ represent the integrated EHH score for ancestral (A) and derived (D) core alleles; and *E* and *SD* are, respectively, expectation and standard deviation of $\left(\frac{iH{H}_A}{iH{H}_D}\right)$.

**Fig. 1:**
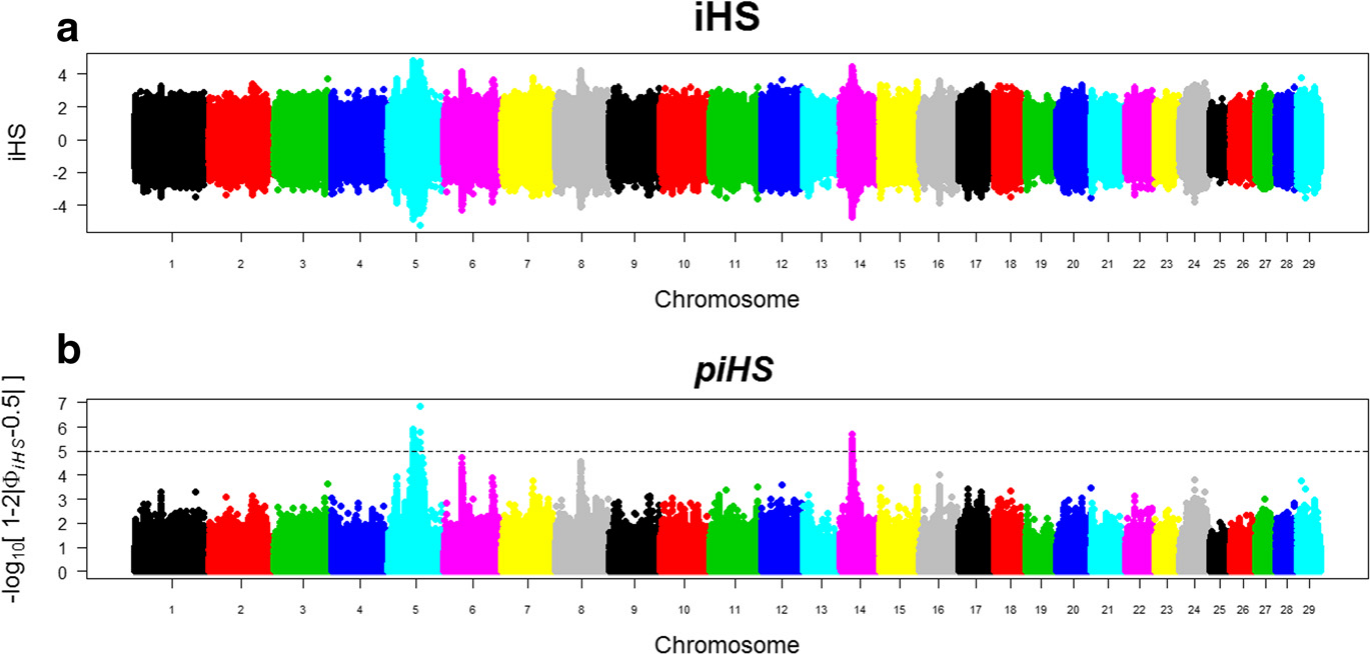
Distribution of integrated haplotype score (iHS) and piHS of each SNP per chromosome. **a** integrated haplotype score (iHS); **b** transformed iHS scores in *piHS* = − log_10_[1 − 2|Φ_*iHS*_ − 0.5|] wherein Φ_*iHS*_ is the Gaussian cumulative distribution function of iHS. Markers with statistically significant piHS values (*P* < 0.00001) are above the dashed line. Only SNPs with MAF ≥ 0.05 were considered

Values of iHS were standardized so that they followed a standard normal distribution (iHS ~ N (0,1)). Thus, the iHS values of different SNPs are directly comparable, regardless of allelic frequencies of these markers. The rehh package [23] was used to calculate the iHS for each allele (ancestral and derivative). The online database available in the supplementary material of Utsunomiya et al. [24] was used as source to determine the ancestor alleles. These authors defined the ancestral alleles using genotyped data of species considered common founders of the Bovinae subfamily *(Bos gaurus, Bubalus bubalis* and *Bos grunniens)*, in which the alleles were fixed at MAF equal to zero. Furthermore, SNPs with lower MAF represents alleles near fixation (i.e. background ancestry) [24]. Therefore, these are not useful for the iHS method, as this enables identifying only recent selection. Thus, SNPs with MAF greater than or equal to 0.05 were used to represent the selection signature regions in Canchim cattle (Fig. 1).

The iHS statistical values were transformed into *piHS* = − log_10_[1 − 2|Φ_*iHS*_ − 0.5|] in which Φ_*iHS*_ is the cumulative distribution Gaussian function of iHS. This transformation allows better visualization and comparison of regions with selection signatures [25]. Assuming that iHS statistics are normally distributed, piHS can be interpreted as ${ \log}_{10}\frac{1}{P}$, in which *P* is the *P*-value associated with the null hypothesis (no selection). Values of piHS greater than or equal to five were considered statistically significant (*P* <0.00001), thus rejecting the null hypothesis.

The linkage disequilibrium between genetic markers was obtained for the SNPs within selection signature regions by means of the r^2^ measure proposed by Hill and Robertson [22]. The software plink [26] was used to conduct the linkage disequilibrium analyses.

### Survey for associated genes

The SNPs with statistically significant iHS (*P* < 0.00001) were inspected using the National Center for Biotechnology Information [27] and Ensembl Genome Browser [28] databases to identify genes or surrounding genes. Panther [29] and DAVID 6.7 [30, 31] databases were used to survey biological processes and metabolic pathways in which the identified genes are involved.

For the candidate selection regions, statistically significant (*P* < 0.00001) iHS values were computed across the genome and averaged within non-overlapping windows of 500 kb. The QTL Animal database [32] was used to identify quantitative trait loci (QTL) described in the literature for each window.

## Results

After the QC, a total of 396 animals and 687,655 SNPs remained for the selection signature study. Fixed alleles in the population (3,270 SNPs) were excluded from the analysis. The iHS distribution was approximately normal (iHS ~ N (0,1)), thus the markers and the chromosomes can be compared. In Fig. 1a are presented the dispersion of the iHS by chromosome, while in Fig. 1b the same markers are presented transformed into piHS.

**Fig. 2:**
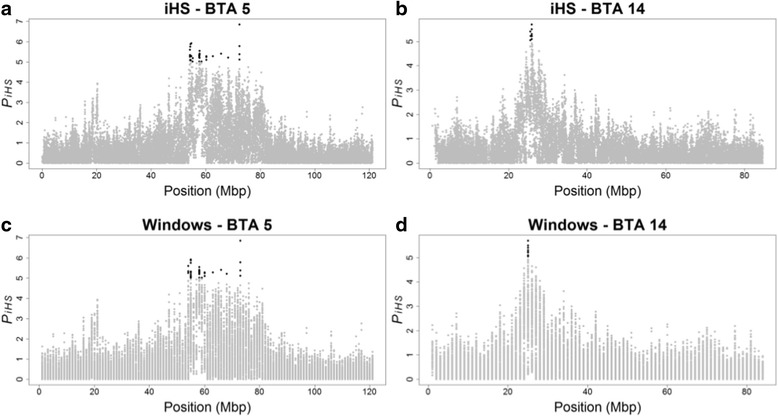
Values of piHS for SNPs (**a** and **b**) and windows (**c** and **d**) on BTA5 and BTA14. Statistically significant regions (*P* < 0.00001), respective positions in base pairs (bp), with statistically significant SNPs and windows (*P* < 0.00001) highlighted in black

The statistically significant regions (*P* < 0.00001) were identified on BTA5 and BTA14 (Fig. 1b). In Fig. 2 are presented the distributions of piHS for SNPs (Fig. 2a, b) and windows (Fig. 2c, d) on BTA5 and BTA14. In Table 1 are presented the descriptive statistics for BTA5 and BTA14 according to SNPs and windows. Despite being distributed in nine windows, the SNPs on BTA5 are concentrated in a region of about 18 Mb (between the 54,155,228 bp and 72,410,862 bp positions), which represents 15 % of the chromosome total size. On BTA14, all significant SNPs (*P* < 0.00001) are concentrated in an interval of only 1 Mb (window 25).

**Table 1:** Descriptive statistics of selection of signatures regions on BTA5 and BTA14. Informative values related to autosomal chromosomes that showed significant SNPs (*P* < 0.00001) and windows

Descriptive statistics	Autosomal chromosomes	
	BTA 5	BTA 14
Length, bp	121,151,689	84,034,538
Total SNPs	30,702	21,745
Number of windows	121	84
Average SNPs/window	253.75	258.87
Number of significant SNPs (Number of windows)	39 (9)	9 (1)

There were 39 and nine significant SNPs found on BTA5 and BTA14, respectively (Table 2). In order to identify selection signature regions which could be associated to any of the phenotypic traits used in the traditional selection, we considered the significance level of *P* < 0.00001 for piHS. None of the identified SNPs were located in protein coding regions, 17 SNPs were observed in intronic regions, one in 3′ untranslated region (UTR), one in 5′ UTR, and 20 in intergenic regions of the BTA5. All SNPs of the BTA14 were mapped into intergenic regions.

**Table 2:** Statistically significant SNPs (*P* < 0.00001) in selection signature regions

dbSNP ^a^	BTA ^b^	Pos, bp^c^	SNP ^d^	iHS ^e^	piHS ^f^	Window	Gene	Distance gene-SNP ^g^	Region
rs109140890	5	54,155,228	[A/G]	4.71	5.61	54	*SLC16A7*	0	Intron
rs109099698	5	54,195,124	[A/G]	4.54	5.26	54	*SLC16A7*	0	Intron
rs134749225	5	54,198,119	[A/G]	−4.58	5.34	54	*SLC16A7*	0	Intron
rs137138760	5	54,272,467	[G/T]	−4.47	5.11	55	*LOC101907520*	33,220	Intergenic
rs109120533	5	54,288,524	[C/T]	4.57	5.32	55	*LOC101907520*	49,277	Intergenic
rs135504287	5	54,295,924	[A/C]	−4.78	5.76	55	*LOC101907520*	56,677	Intergenic
rs133591560	5	54,350,533	[C/T]	−4.46	5.08	55	*LOC101907520*	111,286	Intergenic
rs111002641	5	54,379,176	[A/C]	4.84	5.89	55	*LOC101907520*	139,929	Intergenic
rs133087713	5	54,399,438	[A/G]	−4.57	5.32	55	*LOC101907520*	160,191	Intergenic
rs135847398	5	54,511,415	[C/T]	−4.46	5.09	55	*LOC101907520*	272,168	Intergenic
rs110300059	5	54,745,039	[C/T]	−4.56	5.29	55	*LRIG3*	139,355	Intergenic
rs133256279	5	54,747,486	[A/C]	−4.86	5.93	55	*LRIG3*	136,908	Intergenic
rs132685585	5	55,175,674	[G/T]	−4.43	5.03	55	*LRIG3*	239,313	Intergenic
rs136462146	5	55,199,380	[C/T]	−4.51	5.19	55	*LRIG3*	263,019	Intergenic
rs108956573	5	57,606,624	[C/T]	−4.58	5.33	58	*RPS26*	0	5′ UTR
rs41657485	5	57,625,533	[A/G]	−4.55	5.28	58	*IKZF4*	0	Intron
rs134894252	5	57,634,601	[A/C]	−4.59	5.35	58	*IKZF4*	0	Intron
rs135485666	5	57,643,602	[C/T]	4.43	5.02	58	*SUOX*	0	Intron
rs134235538	5	57,647,574	[C/T]	−4.55	5.27	58	*RAB5B*	0	3′ UTR
rs137809406	5	57,679,838	[C/T]	4.52	5.22	58	*DGKA*	0	Intron
rs29018280	5	57,681,031	[G/T]	4.61	5.40	58	*DGKA*	0	Intron
rs135598509	5	57,690,096	[C/T]	−4.60	5.37	58	*DGKA*	0	Intron
rs134140651	5	57,697,408	[C/T]	−4.52	5.21	58	*DGKA*	0	Intron
rs137073278	5	57,710,890	[C/T]	−4.59	5.36	58	*PYM1*	0	Intron
rs135368690	5	57,729,851	[A/C]	−4.59	5.36	58	*PYM1*	5,697	Intergenic
rs133170163	5	57,736,703	[C/T]	−4.59	5.36	58	*PYM1*	12,552	Intergenic
rs134681832	5	57,741,701	[C/T]	−4.68	5.55	58	*PYM1*	17,550	Intergenic
rs135835510	5	58,467,329	[A/G]	−4.43	5.02	59	*LOC782296*	2,533	Intergenic
rs109346532	5	60,260,395	[C/T]	−4.47	5.11	60	*TESPA1*	5,391	Intergenic
rs134633547	5	60,261,561	[C/T]	−4.47	5.11	60	*TESPA1*	4,225	Intergenic
rs109158476	5	60,263,049	[C/T]	4.56	5.28	60	*TESPA1*	2,737	Intergenic
rs134963725	5	60,267,575	[A/G]	4.54	5.25	60	*TESPA1*	0	Intron
rs29002144	5	62,646,555	[C/T]	−4.55	5.28	63	*LOC101905642*	253,100	Intergenic
rs108993286	5	65,656,474	[A/G]	4.62	5.42	66	*SPIC*	5,989	Intergenic
rs109866300	5	68,257,487	[A/G]	4.52	5.22	68	*TXNRD1*	0	Intron
rs110636438	5	72,348,422	[C/T]	4.61	5.39	73	*LARGE*	0	Intron
rs110912484	5	72,353,301	[A/G]	4.79	5.79	73	*LARGE*	0	Intron
rs41669840	5	72,405,286	[G/T]	−5.26	6.85	73	*LARGE*	0	Intron
rs136536638	5	72,410,862	[A/C]	−4.47	5.11	73	*LARGE*	0	Intron
rs137267491	14	25,505,663	[G/T]	4.44	5.05	25	*IMPAD1*	39,242	Intergenic
rs41627946	14	25,506,575	[G/T]	−4.62	5.41	25	*IMPAD1*	38,330	Intergenic
rs134846474	14	25,537,252	[A/G]	−4.53	5.22	25	*IMPAD1*	7,653	Intergenic
rs137748068	14	25,819,872	[A/G]	−4.47	5.10	25	*FAM110B*	230,370	Intergenic
rs42299083	14	25,846,511	[C/T]	−4.52	5.22	25	*FAM110B*	203,731	Intergenic
rs42299080	14	25,851,646	[C/T]	−4.75	5.70	25	*FAM110B*	198,596	Intergenic
rs136141080	14	25,863,924	[C/T]	−4.66	5.50	25	*FAM110B*	186,318	Intergenic
rs133252286	14	25,866,853	[C/T]	−4.54	5.24	25	*FAM110B*	183,389	Intergenic
rs134567839	14	25,877,586	[C/T]	−4.57	5.31	25	*FAM110B*	172,656	Intergenic

^a^ dbSNP, SNP reference name

^b^ BTA, *Bos taurus* autosome

^c^ Pos, position in base pairs (bp) according to the Bovine UMD3.1 assembly

^d^ SNP, Single Nucleotide Polymorphism

^e^ iHS, integrated haplotype score

^f^ piHS, transformed integrated haplotype score

^g^ The value 0 means that the SNP is located within the gene

The analysis using Panther database identified biological processes related to apoptotic process (GO:0006915), biological adhesion (GO:0022610), biological regulation (GO:0065007), cellular component organization or biogenesis (GO:0071840), cellular process (GO:0009987), developmental process (GO:0032502), immune system process (GO:0002376), localization (GO:0051179), metabolic process (GO:0008152), multicellular organismal process (GO:0032501), and response to stimulus (GO:0050896).

Candidate regions located in windows presented 14 genes and one pseudogene (Table 3). The largest candidate region was observed in window 55 of BTA5, with 11 significant SNPs, two genes (*LRIG3* and *LOC101907520*), and one pseudogene (*LOC785078*) were identified. The smallest candidate region, also on BTA5, totaled 7,180 bp and had only one annotated gene (*TESPA1*). The region with the most of the significant SNPs (13 markers) was observed in window 58 of BTA5 and presented eight genes (*IKZF4*, *DGKA*, *RAB5B*, *PYM1*, *SUOX*, *PMEL*, *CDK2*, and *RPS26*).

**Table 3:** Description of selection signatures and genes identified in candidate regions

Candidate region ^a^	Window	SNP ^b^	Genes
5:5,415,5228…5,4198,119 (42.9 Kb)	54	rs109140890, rs109099698, rs134749225	*SLC16A7*
5:54,272,467…55,199,380 (926.9 Kb)	55	rs137138760, rs109120533, rs135504287, rs133591560, rs111002641, rs133087713, rs135847398, rs110300059, rs133256279, rs132685585, rs136462146	*LRIG3, LOC785078, LOC101907520*
5:57,606,624…57,741,701 (135.1 Kb)	58	rs108956573, rs41657485, rs134894252, rs135485666, rs134235538, rs137809406, rs29018280, rs135598509, rs134140651, rs137073278, rs135368690, rs133170163, rs134681832	*IKZF4, DGKA, RAB5B, PYM1, SUOX, PMEL, CDK2, RPS26*
5:60,260,395…60,267,575 (7.2 Kb)	60	rs109346532, rs134633547, rs109158476, rs134963725	*TESPA1*
5:72,348,422…72,410,862 (62.4 Kb)	73	rs110636438, rs110912484, rs41669840, rs136536638	*LARGE*
14:25,505,663…25,877,586 (371.9 Kb)	25	rs137267491, rs41627946, rs134846474, rs137748068, rs42299083, rs42299080, rs136141080, rs133252286, rs134567839	*IMPAD1*

^a^ Chromosome: position of the first and last statistically significant SNP (*P* < 0.00001) within the window

^b^ SNP: Single Nucleotide Polymorphism

The windows 55 (11 SNPs) and 58 (13 SNPs) identified on BTA5, were located in QTL (quantitative trait loci) regions reported for resistance to ticks, fat thickness, marbling, body weight at birth and yearling, preweaning and average daily gain, and calving ease [33–40]. In the candidate region on BTA14 (window 25), between 25,505,663 and 25,877,586 bp, QTL were reported as associated with resistance to ticks, carcass weight, fat thickness and ribeye area, birth weight and average body weight, average pre-weaning daily gain, calving ease, and gestational age [39–44].

The SNPs with piHS with significance of *P* < 0.0001 are shown in Additional file 1. In this significance level, 296 statistically significant SNPs (*P* < 0. 0001) were observed covering 24, three, two, five and one windows on BTA5, BTA6, BTA8, BTA14, and BTA16, respectively. In the candidate regions, between the first and last significant SNP of these 35 windows, 167 genes or pseudogenes were identified. There are 157 genes or pseudogenes in the BTA5 candidate regions, five in BTA8 regions, four in BTA14 regions and one in the BTA16 region.

Pathway analyses for the genes presented in the Additional file 1 revealed androgen/estrogene/progesterone biosynthesis, gonadotropin releasing hormone receptor pathway, fibroblast growth factor (FGF) signaling pathway, DNA replication, integrin signaling pathway, B cell activation, T cell activation, interleukin signaling pathway, inflammation mediated by chemokine and cytokine signaling pathway, endocytosis, cell cycle, oocyte meiosis, progesterone-mediated oocyte maturation, among others.

The average linkage disequilibrium on BTA5 and BTA14 were equal to 0.65 and 0.52, respectively. The extent of the linkage disequilibrium in each of the candidate selection regions are presented in the Additional file 2. According to Pérez O’Brien et al. [45] high linkage disequilibrium over an extended region is likely the result of recent selection.

## Discussion

The selection signatures herein identified are located in important QTL regions of beef cattle, which includes productive (birth, weaning and yearling weights), reproductive, and conformation traits. These traits are also included in the Canchim selection index. Machado et al. [3] found in Canchim cattle, QTL associated with birth weight and body weight at 365 days old. Genome-wide association for scrotal circumference measured at long yearling age was identified on BTA5, between 45.24 and 45.35 Mb, in Canchim cattle and between 41.98 and 78.59 Mb on BTA14 [46].

In a study by Bolormaa et al. [47], genome-wide association on BTA5 and BTA14 were observed for stature, fatness, and reproduction in beef cattle using multi-trait model. These authors used taurine and zebu breeds and composite animals in the study. Pérez O’Brien et al. [45] used methodology based on regions with lower genetic variability and specific regional linkage disequilibrium patterns to the study selection signatures in Nelore, Gyr, Angus and Brown Swiss breeds. These authors observed genes with potential adaptive and productive importance on BTA5 and BTA14. The BTA14 has been widely explored for genes related to important economic traits in beef and dairy cattle [48].

The iHS statistic gives an idea of how unusual haplotypes around a given SNP are, relative to the genome as a whole, or how different the selected region is in relation to a region subject to the consequences of random mating in the population. Thus, iHS values equal or near zero indicate EHH decay rates similar to ancestral and derivatives core alleles, negative values indicate long haplotypes around a derivative core allele, and positive values indicate long haplotypes around an ancestral core allele, as seen in Fig. 1a. Both iHS extremes are indicative of conserved regions, due to direct selection on derivative alleles, and hitchhike effect, caused by the linkage disequilibrium between ancestral alleles with selected regions [20]. Highly conserved regions over the recent generations in this population are concentrated on the observed peaks (signals) (Fig. 1b). Thus, it is presumed that highly conserved regions are maintained through selection favoring this population.

Kemper et al. [49] used methodologies based on the haplotype homozygosity and found evidence of selection in regions on BTA5, BTA14 and BTA16 in Charolais animals, which corroborate with the results of our study. On BTA5 and BTA14, these authors found selection signatures between 52.8 and 64.75 Mb and 19.75 and 29.55 Mb, respectively. Somavilla et al. [50] investigated selection signatures in Nelore animals using the EHH methodology and verified significant regions in the *FABP4* gene (fatty acid binding protein 4, adipocyte) located on BTA14, this gene plays important role in lipid metabolism and adipocytes homeostasis [51], while the SNPs in this gene have been described as associated with marbling and subcutaneous fat deposit in Wagyu × Limousin F2 crosses [52] and intramuscular fat levels in crossbred *Bos taurus* cattle [53]. In our study, the *FABP4* gene was not found in the candidate regions, perhaps because of the significance level (*P* < 0.00001) considered and sliding windows approach.

The selection conducted in Canchim has transmitted and preserved some regions of the founder breeds across generations. Utsunomiya et al. [24] found regions of recent selection signatures on BTA1, BTA2, and BTA18. We can assume that, as the genetic proportion of zebu is low in the formation of Canchim cattle, greater resemblance of Canchim with Charolais are expected. According to Porto-Neto et al. [54], the linkage disequilibrium is higher in taurine than in zebu cattle. This fact corroborates the results observed in our study, because adjacent loci in greater linkage disequilibrium (probably from the Charolais breed contribution) provide more extended and, apparently, more conserved haplotypes among Canchim and Charolais animals. The majority of detected signals may have arisen during breed formation [24] and large conserved regions could also refer to the selection that occurred in the founder breeds of Canchim.

The region on BTA5 harbored many genes including *PMEL, SUOX, SLC16A7, TESPA1, SPIC, RAB5B, DGKA, PYM1, TXNRD1,* and *LARGE.* These genes are involved in biological functions, such as coat color, amino acids metabolism, transport of glucose, development of immune cells, glicerolipids and phospholipids metabolism, fatty acids metabolism, and biosynthesis of glycoproteins.

The *PMEL* (premelanosome protein) gene, identified in the candidate region of window 58 on BTA5, is involved in the melanin biosynthesis process, in which the primary function is the pigmentation of hair, skin, eyes, and mucous membranes. According to Gutiérrez-Gil et al. [55], mutations described in this gene are partly responsible for the dilution of pigmentation in Charolais cattle, resulting in the white colored coat of the breed. This white coat is an important trait of Charolais cattle and this breed standard is retained in Canchim, through the selection of animals with light colored coat. The light colored coat used to characterize the Canchim, may have conserved the mutation for pigment dilution, resulting in selection of the alleles on *PMEL* gene and extended conservation to gene neighboring regions, due to linkage disequilibrium. This fact can be seen in this study, in which the most significant SNPs in window 58 on BTA5 had negative iHS values (10 out of 13 SNPs). Thus, core alleles are derivative alleles, i.e., originating from migration or gene mutation.

The *TESPA1* (thymocyte expressed, positive selection associated 1) gene in the window 60 and *SPIC* (Spi-C transcription factor) gene in the window 66, are involved in the development of immune cells, related to innate immunity (red pulp macrophages development - [56] and acquired immunity (T-cell development - [57]; and B cells differentiation - [58]). Genes of the immune system are often related to positive selection due to the continued resistance to pathogens [59]. The zebu breeds, crossbreds animals, and composite breeds are characterized by resistance to endo- and ectoparasites [39] and, therefore, it is speculated that the identified selection signatures associated with the immune system genes reflects these selected traits.

On BTA14, we identified the *IMPAD1* (inositol monophosphatase domain containing 1) gene that plays a role in the bone-cartilage system. This gene participates in sulfur and inositol phosphate metabolism, and mutations identified in this gene are associated with chondrodysplasia and abnormal joint development in humans [60]. In a genome-wide association study, Fortes et al. [61] observed association of SNPs in this gene with age at detection of the first corpus luteum and scrotal circumference in Brahman cattle. In Canchim cattle, selection has been practiced for scrotal circumference due to its association with reproductive and productive performance and because of its pleiotropic effect with female reproductive traits [7].

Recent studies have identified selection signature in a candidate region on BTA14, which comprises the *PLAG1* gene [62, 63]. This gene has association with stature and body weight [64], reproductive [65], and carcass traits [66] in cattle. In our study, we identified two regions (24,425,758–24,473,841 bp and 25,505,663–25,987,996 bp) on BTA14 surrounding the *PLAG1* gene region (25,555,459–25,052,403 bp). We believe that *PLAG1* was not observed in the windows herein determined due to methodological differences and different sliding windows approach in the consulted literature. Considering the associations of *PLAG1* gene described in the literature and the proximity to the candidate regions observed in our study, this gene could be an interesting target of selection in Canchim beef cattle.

## Conclusion

The selection signature regions were detected in Canchim cattle, suggesting that the selection processes conducted in this breed (human driven selection during breed formation) and its founders led to the genomic conservation in some regions on BTA5 and BTA14. These selection signatures regions could be associated with Canchim characterization, which sought to unite desirable characteristics of zebu and taurine breeds.

## Additional files

Additional file 1: Candidate regions identified for piHS = 4 (*P* < 0.0001). (DOC 61 kb) Additional file 2: Extent of linkage disequilibrium in candidate selection regions present on BTA5 (A) and BTA14 (B). (TIFF 1970 kb)

## Abbreviations

Bp: base pairs
BTA: *Bos taurus* chromosome
EHH: extended haplotype homozygosity
iHS: integrated haplotype score
Kb: kilobase
Mb: megabase pairs
QC: quality control
QTL: quantitative trait loci
SNP: single nucleotide polymorphism
SS: selection signature
