# Supplementary material for: Selection signatures in Canchim beef cattle
Source: J Anim Sci Biotechnol. 2016 May 5;7:29. doi: 10.1186/s40104-016-0089-5 (PMC4858954; doi:10.1186/s40104-016-0089-5)
Supplement: Additional file 1: — Candidate regions identified for piHS = 4 (P < 0.0001). (DOC 61 kb) [file 40104_2016_89_MOESM1_ESM.doc]

**Additional file 1** Candidate regions identified for piHS = 4 (*P* < 0.0001)

| **Candidate region Chr:Pos^1^** | | **Window** | **Statistically significant SNPs (*P* < 0.0001)** | **Genes in candidate regions** |
| --- | --- | --- | --- | --- |
| **5:**46,485,163 | | 47 | rs41670148 | intergenic region |
| **5:**51,031,728 | | 51 | rs137541516 | *PPM1H* |
| **5:**53,828,254…54,205,948 (377.7 Kb) | | 54 | rs136875231, rs135675226, rs132860195, rs136406643, rs110196892, rs134317802, rs137662784, rs109140890, rs137096832, rs109099698, rs134749225, rs133184506 | *SLC16A7* |
| **5:**54,246,704…55,199,380 (952.6 Kb) | | 55 | rs135855458, rs133607360, rs137138760, rs109120533, rs135504287, rs109129425, rs133591560, rs111002641, rs133087713, rs135847398, rs135232465, rs110300059, rs133256279, rs136713461, rs134337764, rs135513869, rs133212739, rs136464884, rs108966774, rs137300782, rs132685585, rs134117357, rs135562579, rs134745660, rs133221235, rs135018237, rs136462146 | *LRIG3, LOC785078* |
| **5:**56,452,644…57,219,551 (766.9 Kb) | | 57 | rs135396753, rs137452746, rs137017835, rs110707337, rs135459297, rs110038969, rs109703343, rs133644295, rs135140806, rs134716056, rs132636761, rs110311254, rs132803146, rs136816142, rs136497465, rs136128750, rs109770307, rs134825518 | *R3HDM2, NACA, SHMT2, BAZ2A, LRP1, ATP5B, STAT6, NDUFA4L2, MYO1A, STAC3, ZBTB39, LOC100336976, SDR9C7, LOC100138638, HSD17B6 PRIM1, LOC784846, PTGES3, RBMS2, NXPH4, MIR2431, NAB2, NEMP1, TAC3, GPR182, RDH16* |
| **5:**57,265,570…58,113,423 (847.8 Kb) | | 58 | rs17871459, rs110765282, rs134525960, rs110441895, rs137018936, rs132802109, rs133029950, rs134101094, rs133329393, rs135222721, rs136871149, rs137760062, rs137186444, rs136605108, rs137336736, rs108956573, rs137486517, rs136907721, rs41657485, rs134894252, rs136110210, rs135485666, rs134235538, rs133560501, rs134487259, rs137809406, rs29018280, rs135598509, rs134140651, rs137489060, rs137073278, rs135368690, rs133170163, rs134681832, rs137639435, rs136136938, rs135419986, rs136277669, rs134842047, rs134513329, rs109554404, rs110044955, rs109333650, rs135822426, rs133332639, rs136702307 | *TIMELESS, STAT2, CS, ANKRD52, RNF41, SMARCC2, ESYT1, ERBB3, IKZF4, DGKA, MMP19, SARNP, ITGA7, OR10P1, OR2AP1, MIP, APOF, MIR2432, COQ10A, NABP2, TRNAS-CGA, MYL6B, PA2G4, RPS26, RAB5B, PYM1, DNAJC14, GDF11, RDH5, METTL7B, OR6C4, APON, PAN2, MIR2433, SLC39A5, MYL6, ZC3H10, SUOX, PMEL, BLOC1S1, IL23A, CNPY2, CDK2, ORMDL2, CD63, LOC507581, LOC101903534, LOC520938, LOC781363, LOC615284, LOC618816* |
| **5:**58,311,209…58,918,146 (606.9 Kb) | | 59 | rs134929599, rs133505213, rs135462297, rs136759922, rs133959751, rs134519015, rs135835510, rs109376165, rs110088220, rs137223708, rs132815847, rs133751107, rs135317911, rs133694247, rs132764104, rs137127954, rs136853443, rs137025170 | *OR6C2, OR6C75, OR6C68, OR6C76, LOC100140445, LOC782296, LOC787518, LOC787535, LOC512580, LOC507560, LOC782430, LOC782338, LOC782220, LOC787659* |
| **5:**59,694,965…60,268,464 (573.5 Kb) | | 60 | rs110701251, rs136102673, rs110800764, rs133645814, rs134598243, rs42917108, rs136226027, rs134907856, rs137435186, rs109294609, rs135511176, rs133124323, rs133047423, rs108980787, rs109346532, rs134633547, rs109158476, rs110695325, rs134963725, rs136426418 | *NEUROD4, TESPA1, OR9K2, LOC788438, LOC788512, LOC100139733, LOC788524, LOC788583, LOC788615, LOC512340, LOC511753, LOC527216, LOC788998, LOC788535, LOC788552* |
| **5:**60,367,377 | | 61 | rs137202845 | intergenic region |
| **5:**62,602,488…62,663,989 (61.5 Kb) | 63 | | rs134204031, rs137211355, rs29003802, rs136715798, rs29002144, rs110565895 | intergenic region |
| **5:**63,391,039…64,071,626 (680.6 Kb) | 64 | | rs136879411, rs137612005, rs133055198, rs134425569, rs135767466, rs135538191, rs133438080, rs41575595, rs136689600, rs110412352 | *ANKS1B* |
| **5:**64,404,709…65,087,060 (682.3 Kb) | 65 | | rs133336046, rs135193455, rs134601011, rs135886485, rs132646565, rs135822742, rs110989538, rs136034421, rs132649255, rs137188875, rs136419402 | *UHRF1BP1L, SCYL2, SLC17A8, NR1H4, ACTR6, GAS2L3, ANO4, MIR2434* |
| **5:**65,577,118...66,083,568 (506.4 Kb) | 66 | | rs134081668, rs108993286, rs41604581, rs41604578, rs110304399, rs41660455 | *UTP20, SPIC, ARL1, MYBPC1, SYCP3, GNPTAB, CHPT1, DRAM1, LOC101902002, LOC101902154* |
| **5:**68,213,492…68,257,487 (44.0 Kb) | 68 | | rs109203752, rs133116549, rs110661962, rs110987778, rs133296950, rs134651571, rs109866300 | *TXNRD1, EID3* |
| **5:**68,335,096…68,592,141 (257.0 Kb) | 69 | | rs110701570, rs110524777, rs110363346, rs134490863, rs109583269, rs110980449, rs133528932, rs135824832 | *CHST11* |
| **5:**69,557,275 | 70 | | rs133616493 | intergenic region |
| **5:**71,219,751…71,223,955 (4.2 Kb) | 71 | | rs134256760, rs110963272, rs137277598 | *BTBD11* |
| **5:**72,006,885…72,298,266 (291.4 Kb) | 72 | | rs110794743, rs41257131, rs110227071, rs134667803, rs135858044, rs41627002, rs136695775, rs134438472, rs136121594 | *MGC137211, MGC137014, LARGE, LOC511240* |
| **5:**72,345,409…72,4421,19 (96.7 Kb) | | 73 | rs41572821, rs110636438, rs110912484, rs110272161, rs41669840, rs136536638, rs42570409 | *LARGE* |
| **5:**74,199,996 | | 74 | rs133941437 | intergenic region |
| **5:**74,373,230 | | 75 | rs137468844 | *RBFOX2* |
| **5:**75,356,941…76,049,319 (692.4 Kb) | | 76 | rs110278932, rs109796974, rs43442275, rs137785235, rs110628104 | *CACNG2, IFT27, PVALB, NCF4, CSF2RB, TEX33, MPST, TST, TMPRSS6, KCTD17, C1QTNF6, RAC2, SSTR3, LOC510185* |
| **5:**78,446,197 | | 79 | rs133080391 | intergenic region |
| **5:**80,411,994 | | 81 | rs109880277 | *TMTC1* |
| **6:**38,471,732 | | 38 | rs109547063 | intergenic region |
| **6:**39,224,898…39,537,122 (312.2 Kb) | | 39 | rs135215113, rs137167351, rs135351468, rs108950522, rs110350585, rs110009223, rs136490720, rs42820832, rs110497358 | intergenic region |
| **6:**40,000,025 | | 40 | rs132777479 | intergenic region |
| **8:**52,178,705…52,180,527 (1.8 Kb) | | 52 | rs43557964, rs41655539 | intergenic region |
| **8:**52,191,836…52,996,466 (804.6 Kb) | | 53 | rs43557959, rs43557958, rs43557949, rs43557940, rs109883376, rs109516431, rs109435236, rs135898479, rs43550796 | *LOC528098, RFK, GCNT1, PRUNE2, LOC101908092* |
| **14:**24,425,758…24,473,841 (48.1 Kb) | | 24 | rs109185321, rs110717761, rs135958550, rs110543321, rs42646635 | *XKR4* |
| **14:**25,505,663…25,987,996 (482.3 Kb) | | 25 | rs137267491, rs41627946, rs136889989, rs133736127, rs134846474, rs43770985, rs135744414, rs137748068, rs137494880, rs42299083, rs134006862, rs42299080, rs134601995, rs136141080, rs133252286, rs135734725, rs136755107, rs134567839, rs136146069, rs134624153, rs41665273, rs42298501, rs42298505, rs110721536, rs42298481, rs42298477, rs109372952, rs42298467, rs29017100 | *IMPAD1* |
| **14:**26,003,598…26,226,856 (223.2 Kb) | | 26 | rs110267284, rs110118008, rs109826093, rs42305732, rs136470858, rs110784513, rs135249313, rs42304759, rs42304742 | *FAM110B* |
| **14:**27,669,598…27,785,247 (115.6 Kb) | | 27 | rs109531568, rs41720445, rs110432430, rs41720529, rs29027563, rs109950422, rs133771282 | *CA8* |
| **14:**28,403,939…28,406,454 (2.5 Kb) | | 28 | rs109022234, rs110183859 | intergenic region |
| **16:**45,857,640 | | 46 | rs137064940 | *RERE* |

^1^Chr: chromosome, Pos: position in base pairs
