# Supplementary figures and images for: Selection signatures in Canchim beef cattle
Source: J Anim Sci Biotechnol. 2016 May 5;7:29. doi: 10.1186/s40104-016-0089-5 (PMC4858954; doi:10.1186/s40104-016-0089-5)

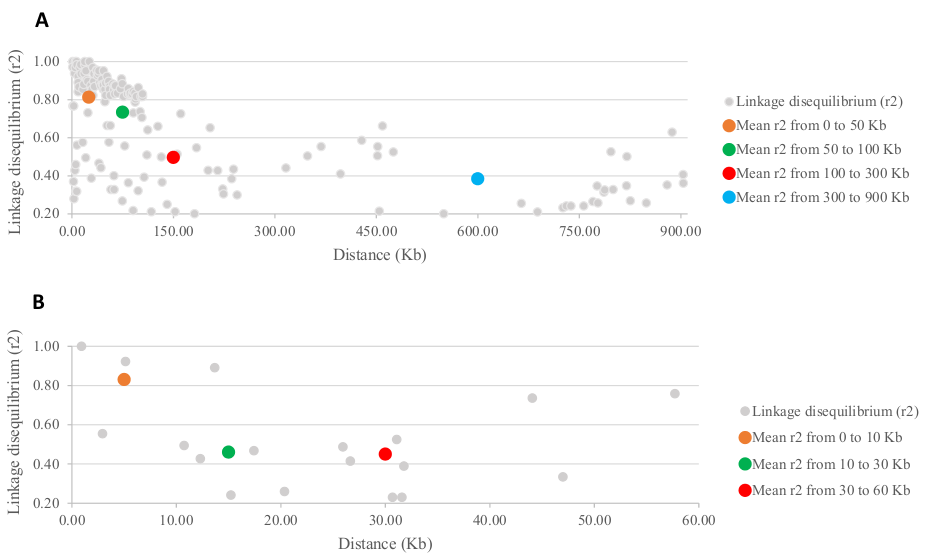

Supplement: Additional file 2: — Extent of linkage disequilibrium in candidate selection regions present on BTA5 (A) and BTA14 (B). (TIFF 1970 kb) [file 40104_2016_89_MOESM2_ESM.tiff]
